# Hongjam, an edible silkworm-derived food, attenuates steatohepatitis and fibrosis via multi-axis modulation of metabolic stress, inflammation, and fibrogenic signaling

**DOI:** 10.3389/fnut.2026.1839551

**Published:** 2026-05-26

**Authors:** Hye-Rin Ahn, Seung-Won Lee, Da-Young Lee, Moon-Young Song, Young-Min Han, Byeong Yeob Jeon, You-Kyung Jang, Eun-Hee Kim

**Affiliations:** 1 College of Pharmacy and Institute of Pharmaceutical Sciences, CHA University, Seongnam, Republic of Korea; 2 QBM Co., Ltd., Seoul, Republic of Korea

**Keywords:** fibrosis, Hongjam, inflammation, lipid metabolism, MASH

## Abstract

**Introduction:**

Metabolic dysfunction-associated steatohepatitis (MASH) is a progressive liver disease characterized by sustained inflammation and fibrosis, for which effective disease-modifying therapies remain limited. In this study, we investigated the hepatoprotective effects of Hongjam, a processed edible silkworm-derived food, in a methionine-choline-deficient (MCD) diet-induced mouse model of MASH.

**Methods:**

Histological analysis, plasma biochemical assessments, western blotting, quantitative PCR analyses, and in vitro experiments using macrophages and HepG2 hepatocyte-derived cells were performed to evaluate the effects of Hongjam and silk fibroin peptides associated with Hongjam.

**Results:**

Hongjam markedly alleviated hepatic injury, as evidenced by improved histological features, normalization of plasma biochemical parameters, and reduced collagen deposition. Mechanistically, Hongjam suppressed activation of the TGF-β/Smad signaling pathway, inhibited NF-κB-mediated inflammatory signaling, and attenuated MCD-associated metabolic stress signaling, including changes in AMPK phosphorylation and the expression of genes involved in fatty acid oxidation and lipid transport. Consistent with the in vivo findings, silk fibroin peptides associated with Hongjam attenuated NF-κB-mediated inflammatory signaling in macrophages and modulated TGF-β/Smad-associated signaling in HepG2 hepatocyte-derived cells in vitro

**Discussion:**

Collectively, these results demonstrate that Hongjam attenuates experimental MASH by concurrently modulating metabolic stress, inflammation, and fibrotic remodeling. These findings highlight Hongjam as a food-derived candidate with potential relevance for dietary strategies targeting metabolic liver diseases.

## 1 Introduction

Metabolic dysfunction-associated steatohepatitis (MASH) represents the progressive inflammatory subtype of metabolic dysfunction-associated steatotic liver disease (MASLD), pathologically defined by lobular inflammation, hepatic steatosis, and varying degrees of fibrosis (1, 2). As a major contributor to chronic liver pathology, MASH can progress to cirrhosis, hepatic decompensation, or hepatocellular carcinoma (HCC), posing a growing threat to global health (3). Its increasing prevalence parallels the global rise in obesity, insulin resistance, and metabolic syndrome. MASLD affects nearly 25% of the global population, with 20–30% progressing to MASH (4, 5). The mechanistic underpinnings of MASH are multifactorial, with key contributions from lipid metabolism dysregulation, mitochondrial dysfunction, oxidative stress, immune activation, and fibrosis (6). According to the “multiple-hit” model, these factors do not act in isolation but rather converge synergistically to drive disease progression from simple steatosis to fibrotic MASH (7–10). Despite recent regulatory approvals of resmetirom (Rezdiffra) and semaglutide (Wegovy), current therapies for MASH remain non-curative, underscoring the urgent need for effective treatments capable of halting or reversing disease progression (11).

In MASH, metabolic stress, inflammatory signaling, and fibrotic remodeling are tightly interconnected, forming a self-reinforcing pathological network rather than linear, independent pathways (12). Consequently, therapeutic strategies that simply activate or inhibit a single signaling axis may be insufficient to effectively restrain disease progression. AMP-activated protein kinase (AMPK) is an important metabolic regulatory component involved in hepatic lipid metabolism, and its phosphorylation can reflect cellular stress under energy-deficient conditions (13). In the methionine-choline-deficient (MCD) diet model, increased hepatic AMPK phosphorylation has been reported previously and may reflect an energy stress response associated with impaired fatty acid oxidation, mitochondrial injury, and ATP depletion (14). This metabolic stress, in turn, acts as a potent trigger for inflammatory signaling through activation of the nuclear factor-κB (NF-κB) pathway, leading to amplified expression of pro-inflammatory cytokines (15). Persistent inflammation further engages transforming growth factor-ꞵ (TGF-ꞵ)/Smad signaling, accelerating fibrotic remodeling and extracellular matrix deposition (16). Thus, metabolic stress, NF-κB-driven inflammation, and TGF-β/Smad signaling are closely interconnected during MASH progression (12).

Hongjam is an edible, processed silkworm-derived preparation in which mature larvae are rendered suitable for dietary use through steaming, followed by freeze-drying and grinding (17, 18). Hongjam is characterized by its high content of silk fibroin peptides and amino acids as major protein-associated constituents (19). Given the liver’s central role in amino acid metabolism, protein-derived nutrients such as amino acids and bioactive peptides may be particularly relevant to hepatic metabolic homeostasis and liver health (20). Based on this rationale, we hypothesized that Hongjam, which is enriched in silk fibroin peptides, could mitigate key pathological features of MASH. Previously, we demonstrated that Hongjam ameliorates early-stage steatotic liver disease in high-fat diet models primarily by improving lipid metabolism and insulin resistance (17). However, progressive MASH extends beyond simple steatosis and is characterized by sustained inflammation and fibrotic remodeling, including lobular inflammation and bridging fibrosis, which are not adequately reproduced in high-fat diet models (21). The MCD diet promotes hepatic triglyceride accumulation by impairing fatty acid oxidation and VLDL export and is widely used to induce steatohepatitis with prominent necroinflammation and fibrosis (14). Therefore, the MCD diet model was selected to evaluate the anti-inflammatory and anti-fibrotic efficacy of Hongjam in a progressive MASH-like context. Here, we evaluated the therapeutic efficacy of Hongjam in an MCD diet-induced model of MASH and examined silk fibroin peptides, a major component of Hongjam, as putative bioactive components in hepatic and immune cell systems, focusing on AMPK-associated metabolic stress responses, NF-κB-mediated inflammatory signaling, and TGF-β/Smad-driven fibrogenesis.

## 2 Materials and methods

### 2.1 Preparation of Hongjam

Hongjam was prepared as described previously (22). Briefly, mature *Bombyx mori* larvae (white-jade cocoon strain) were harvested on the third day of the fifth instar and steamed at 100 °C for 130 min using an electric steamer (KumSeong Ltd., Bucheon, Korea). The steamed larvae were then freeze-dried for 24 h using a freeze dryer (FDT-8612, Operon Ltd., Gimpo, Korea). The freeze-dried larvae were ground into a fine powder (average particle size ~0.1 mm) by sequential milling using a disc mill and a hammer mill (HM001, Korean Pulverizing Machinery Co. Ltd., Incheon, Korea). The resulting Hongjam powder was stored at −50 °C until use.

Hongjam has been previously characterized by amino acid profiling using ion-exchange HPLC with post-column ninhydrin derivatization, identifying glycine (Gly), serine (Ser), and alanine (Ala) as the most abundant components (17). A summary of the previously reported amino acid composition is provided in Supplementary Table S1.

### 2.2 Animal and experimental design

Four-week-old male C57BL/6J mice were purchased from Orient bio (Seoul, Korea). Animals were housed under controlled environmental conditions (24 °C; 12 h light/dark cycle). After a one-week acclimatization period, the mice were randomly divided into experimental groups (Normal, *n* = 8; MCD, *n* = 8; Hongjam- and Silymarin-treated groups, *n* = 8 per group). The normal group received a standard chow diet, while the MCD group was fed the MCD diet. The Hongjam groups received the MCD diet supplemented with Hongjam at 0.01, 0.1, or 1 g/kg body weight. The positive control group was administered the MCD diet with silymarin at 0.1 g/kg body weight. The experimental period was 8 weeks for all groups. Body weight was recorded weekly throughout the experiment. At the end of the experiment, mice were sacrificed under CO₂ anesthesia. Livers were excised, rinsed in PBS, and weighed. A portion was fixed in 10% neutral buffered formalin for histology analysis; and the remaining samples were stored at −80 °C until further use. All procedures were approved by the IACUC of CHA University (IACUC approval No. 240050) and were conducted in accordance with institutional animal welfare regulations. The animal experiments were reported in accordance with the ARRIVE guidelines.

### 2.3 Biochemical analysis

After the 8-week feeding period, the mice were sacrificed, and blood samples were collected into heparinized tubes. The samples were centrifuged at 3,000 rpm for 15 min at 4 °C, and the supernatants were transferred to fresh tubes for biochemical analysis. Plasma levels of alanine aminotransferase (ALT), aspartate aminotransferase (AST), alkaline phosphatase (ALP), albumin, total bilirubin, and lactate dehydrogenase (LDH) were measured using an automatic analyzer (Hitachi 7600-210, Hitachi High-Technologies Corporation, Tokyo, Japan).

### 2.4 Histological analysis

Liver tissues were fixed in 10% neutral buffered formalin, embedded in paraffin, and sectioned at a thickness of 4 μm. Sections were stained with either hematoxylin and eosin (H&E) or Masson’s trichrome (MT) using standard protocols. For histological evaluation, three stained hepatic tissue sections were examined for each experimental group. From each section, three microscopic fields containing a central vein were captured to ensure anatomical consistency for histological scoring. The severity of liver damage was assessed based on histological changes observed in H&E- and MT-stained sections and graded using a 5-point scale. A score of 1 indicated no detectable abnormalities; scores of 2, 3, 4, and 5 represented minimal (<10%), mild (10–25%), moderate (25–50%), and severe (>50%) involvement, respectively, in terms of both lipid accumulation and fibrotic areas. Scoring was performed independently by five blinded observers using the same standardized criteria.

### 2.5 Silk fibroin peptide synthesis

Synthetic silk peptides corresponding to the fibroin hexapeptide sequence Gly-Ala-Gly-Ala-Gly-Ser (GAGAGS) were custom-synthesized by Peptron Inc. (Daejeon, Korea) using standard Fmoc-based solid-phase peptide synthesis (SPPS) techniques. After cleavage from the resin, crude peptides were purified by reverse-phase high-performance liquid chromatography (RP-HPLC) using a C18 column. Elution was performed with a linear gradient of water and acetonitrile (10–75%, v/v) containing 0.1% trifluoroacetic acid (TFA). The purified peptides were lyophilized and dissolved in distilled water prior to use in experiments.

### 2.6 Cell culture and treatment

Murine RAW 264.7 macrophages (ATCC TIB-71; RRID: CVCL_0493) and human HepG2 hepatocellular carcinoma cells (ATCC HB-8065; RRID: CVCL_0027) were purchased from the Korean Cell Line Bank (KCLB) and maintained in accordance with KCLB’s guidelines. To induce inflammation, RAW 264.7 cells were stimulated with LPS (1 μg/mL; Sigma-Aldrich) for 1 or 6 h. To activate TGF-β/Smad-associated signaling responses, HepG2 cells were treated with TGF-β (10 ng/mL; Sigma-Aldrich) for 24 h.

### 2.7 RNA extraction and quantitative real-time PCR (qRT-PCR)

Liver tissues were ground into a fine powder under liquid nitrogen, and cultured cells were harvested following 24 h of treatment with fibroin peptide and TGF-β. Total RNA was extracted from both liver tissues and cells using TRIzol reagent (Invitrogen, Waltham, MA, USA) according to the manufacturer’s instructions. After addition of chloroform (Sigma-Aldrich, St. Louis, MO, USA) and centrifugation at 13,000×*g* for 15 min at 4 °C, the aqueous phase was collected, and RNA was precipitated with isopropanol. The resulting RNA pellet was washed with DEPC-treated 70% ethanol, air-dried, and dissolved in RNase-free water. RNA concentrations were determined using a NanoDrop One spectrophotometer (Thermo Fisher Scientific, Waltham, MA, USA). Two micrograms of total RNA were reverse transcribed using the Labopass™ cDNA synthesis kit (Cosmogenetech, Seoul, Korea). Quantitative real-time PCR was performed using the ViiA™ 7 Real-Time PCR System (Applied Biosystems, Foster City, CA, USA) and Luna® Universal qPCR Master Mix (New England Biolabs, Beverly, MA, USA). Amplification was conducted for 40 cycles with an annealing temperature of 60–63 °C. Relative gene expression was calculated using the 2^−ΔΔCt^ method, with normalization to 18S rRNA. Primer sequences for target genes are provided in Table 1.

**Table 1:** Primer sequences for qRT-PCR.

Gene	Forward	Reverse
Mouse *α-SMA*	TCCAGCCATCTTTCATTGGGA	CCCCTGACAGGACGTTGTTA
Mouse *COL1A1*	CACCCTCAAGAGCCTGAGTC	GTTCGGGCTGATGTACCAGT
Mouse *TIMP1*	TCTTGGTTCCCTGGCGTACTCT	GTGAGTGTCACTCTCCAGTTTGC
Mouse *CYP4A10*	AGTGTCTCTGCTCTAAGCC	CCCAAAGAACCAGTGAAAAG
Mouse *CYP4A14*	TTGCCAGAATGGAGGATAGG	CAGGAAATTCCACTGGCTGT
Mouse *ACOX1*	CCAATCATGCGATAGTCCTGGC	CTTCAGGTAGCCATTATCCATCTC
Mouse *CD36*	CCTTGGCAACCAACCACAAA	ATCCACCAGTTGCTCCACAC
Mouse *LPL*	ATCGGAGAACTGCTCATGATGA	CGGATCCTCTCGATGACGAA
Mouse *LDLR*	TTCAGTGCCAATCGACTCAC	TGTGACCTTGTGGAACAGGA
Mouse *IL-6*	TACCACTTCACAAGTCGGAGGC	CTGCAAGTGCATCATCGTTGTTC
Mouse *IL-1β*	TGGACCTTCCAGGATGAGGACA	GTTCATCTCGGAGCCTGTAGTG
Mouse *IL-8*	TCAATGCCTGAAGACCCTGCCAA	TGGGTTCTTCCGTTGAGGGACAGC
Mouse *TNF-α*	GGTGCCTATGTCTCAGCCTCTT	GCCATAGAACTGATGAGAGGGAG
Human *TGF-β*	TACCTGAACCCGTGTTGCTCTC	GTTGCTGAGGTATCGCCAGGAA
Human *α-SMA*	CTATGCCTCTGGACGCACAACT	CAGATCCAGACGCATGATGGCA
Human *SNAIL*	GCTGCAGGACTCTAATCCAGA	ATCTCCGGAGGTGGGATG
Human *MMP2*	AGTGAATGACATCTTCACGTTTG	CTTGCAAAGGAGATCAGCAA
Human *TNF-α*	AAAACAACCCTCAGACGCCA	TCCTTTCCAGGGGAGAGAGG
*18S rRNA*	GCAATTATTCCCCATGAACG	GGCCTCACTAAACCATCCAA

### 2.8 Protein extraction and Western blotting analysis

Liver tissues were pulverized under liquid nitrogen, and both liver tissues and cells were lysed in cell lysis buffer (Cell Signaling Technology, #9803) supplemented with protease and phosphatase inhibitors. Lysates were incubated on ice for 10 min and centrifuged at 13,000 rpm for 15 min at 4 °C. The supernatants were collected, and protein concentrations were determined using the Pierce™ BCA Protein Assay Kit (Thermo Fisher Scientific). Equal amounts of protein were separated by 10% SDS-PAGE and transferred to PVDF membranes (Millipore). Membranes were blocked with 3% BSA in PBS-T (0.1% Tween-20) for 1 h at room temperature, followed by overnight incubation with primary antibodies at 4 °C. Details of all primary antibodies are listed in Table 2. After washing with PBS-T, membranes were incubated with HRP-conjugated secondary antibodies for 1 h at room temperature. Protein bands were visualized using ECL reagents and detected with the ImageQuant™ LAS 4000 (GE Healthcare).

**Table 2:** Antibodies for Western blotting.

Antibody	Product no.	Species of origin and supplier
TGF-βRI	Sc-101517	Rabbit monoclonal, Santa Cruz Biotechnology, Inc
p-SMAD2	#3108	Rabbit monoclonal, Cell signaling technology, Inc
p-SMAD3	#9520	Rabbit monoclonal, Cell signaling technology, Inc
SMAD2/3	#5678	Rabbit polyclonal, Cell signaling technology, Inc
α-SMA	ab7817	Mouse monoclonal, Abcam
CoL1A1	sc-293182	Mouse monoclonal, Santa Cruz Biotechnology, Inc
TIMP1	sc-365905	Mouse monoclonal, Santa cruz Biotechnology, Inc
CPT1	sc-393070	Mouse monoclonal, Santa cruz Biotechnology, Inc
p-AMPK	sc-33524	Rabbit polyclonal, Santa Cruz Biotechnology, Inc
AMPK	sc-25792	Rabbit polyclonal, Santa Cruz Biotechnology, Inc
p-p50	sc-271908	Mouse monoclonal, Santa Cruz Biotechnology, Inc
P50	sc-1190	Goat polyclonal, Santa Cruz Biotechnology, Inc
p-p65	#3033	Rabbit monoclonal, Cell signaling technology, Inc
P65	sc-8008	Mouse monoclonal, Santa Cruz Biotechnology, Inc
p-IκBα	#2859	Rabbit polyclonal, Cell signaling technology, Inc
IκBα	#9242	Rabbit polyclonal, Cell signaling technology, Inc
COX2	aa 570–598	Mouse polyclonal, Cayman, Co
iNOS	Sc-7271	Mouse monoclonal, Santa cruz Biotechnology, Inc
β-actin	sc-47778	Mouse monoclonal, Santa cruz Biotechnology, Inc

### 2.9 Statistical analysis

All experiments were performed at least three times independently, and data are presented as mean ± SD. Statistical analyses were performed using GraphPad Prism 5 (GraphPad Software). Group differences were evaluated with one-way ANOVA followed by Tukey’s *post hoc* test. A *p*-value less than 0.05 was considered statistically significant.

## 3 Results

### 3.1 Hongjam alleviates hepatic damage and restores liver function in MCD diet–induced MASH

**Figure 1:**
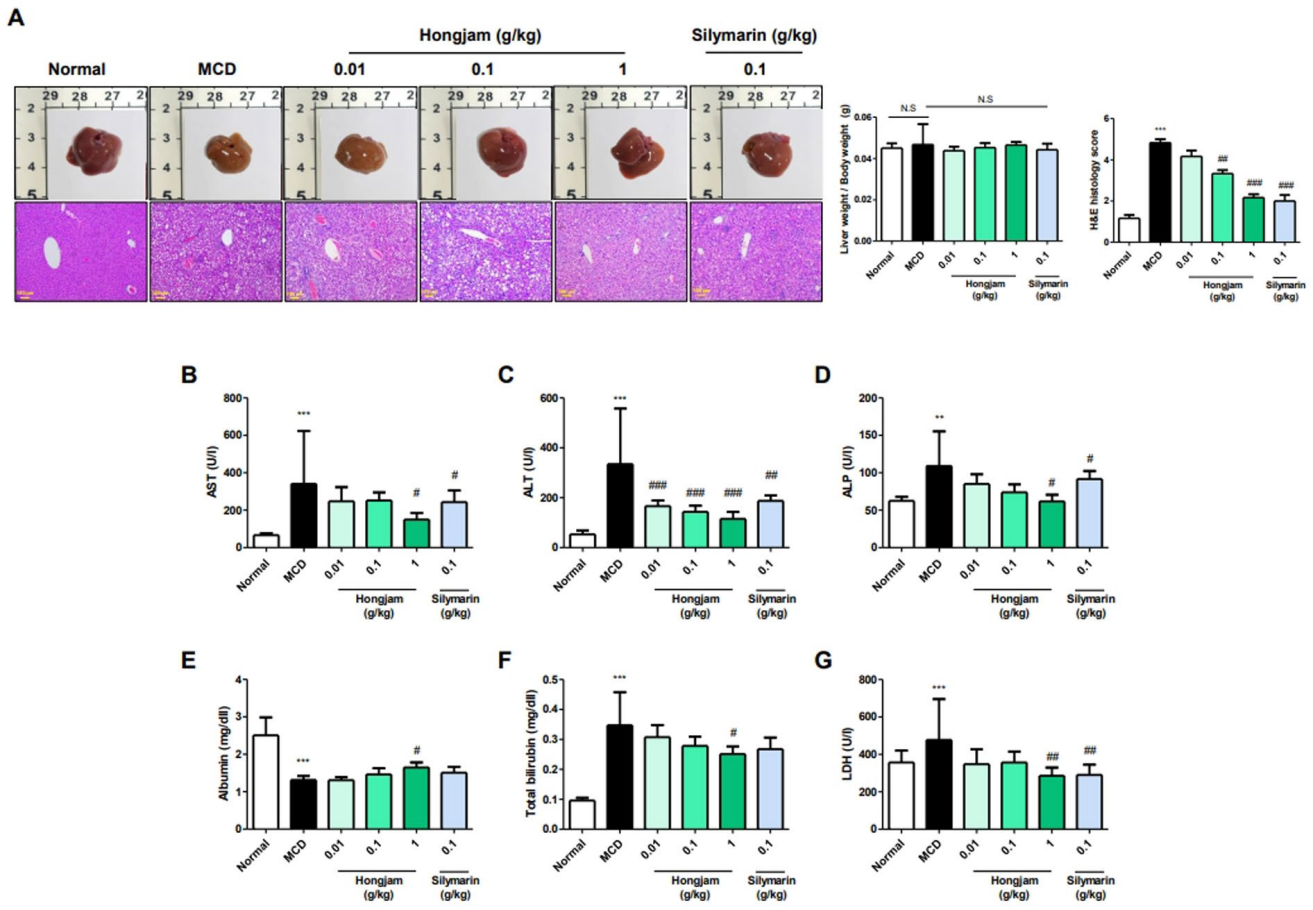
Hongjam alleviates hepatic damage and restores liver function in MCD diet–induced MASH. **(A)** Representative macroscopic liver images and H&E-stained sections show steatosis and architectural disruption in MCD-fed mice, both of which are mitigated by Hongjam (0.01–1 g/kg) or silymarin (0.1 g/kg) treatment (scale bar = 100 μm; original magnification ×100). Liver weight-to-body weight ratios. H&E histological scores. **(B–G)** Plasma levels of AST, ALT, ALP, albumin, total bilirubin, and LDH. Data are presented as mean ± s.d. (*n* = 8 per group). Statistical significance was determined by one-way ANOVA followed by Tukey’s multiple comparisons test. **p < 0.01, ***p < 0.001 vs. normal group; #*p* < 0.05, ##*p* < 0.01, ###*p* < 0.001 vs. MCD group; n.s., not significant.

To evaluate the therapeutic hepatoprotective effects of Hongjam, we employed a methionine–choline-deficient (MCD) diet model of MASH. Gross examination revealed that livers from MCD-fed mice were visibly smaller and exhibited pale, yellowish discoloration compared with those from the normal group, indicative of lipid accumulation and metabolic impairment (Figure 1A). In contrast, livers from mice treated with a high dose of Hongjam (1 g/kg) appeared darker in color and more comparable in size to those of the normal group. Histological liver injury induced by the MCD diet was confirmed by H&E staining, which showed marked hepatic damage in MCD-fed mice (Figure 1A). Consistent with the gross morphological findings, Hongjam treatment markedly attenuated these histopathological alterations, as evidenced by significantly reduced H&E scores (Figure 1A). Although the liver weight-to-body weight ratio did not differ significantly among the MCD-fed groups, histological assessment clearly demonstrated substantial MCD-induced liver injury and its improvement following Hongjam treatment. To further evaluate hepatic injury and the functional effects of Hongjam, plasma biochemical analyses were performed. Plasma levels of AST, ALT, ALP, albumin, total bilirubin, and LDH were measured (Figures 1B–G). The MCD diet markedly increased plasma AST, ALT, ALP, LDH, and total bilirubin levels while significantly reducing albumin levels, reflecting hepatocellular injury, impaired synthetic capacity, and cholestatic stress (23). Hongjam treatment dose-dependently attenuated these biochemical abnormalities. Specifically, plasma AST, ALT, ALP, and LDH levels progressively decreased with increasing doses of Hongjam, whereas albumin levels increased and total bilirubin levels declined. Notably, these effects were most pronounced in the 1 g/kg group and were comparable to those observed in the silymarin-treated group (Figures 1B–G). Collectively, these results demonstrate that Hongjam exerts hepatoprotective activity in MCD-induced MASH, accompanied by broad improvements in biochemical markers of hepatic function.

### 3.2 Hongjam suppresses hepatic fibrosis by targeting the TGF-β/Smad signaling pathway

**Figure 2:**
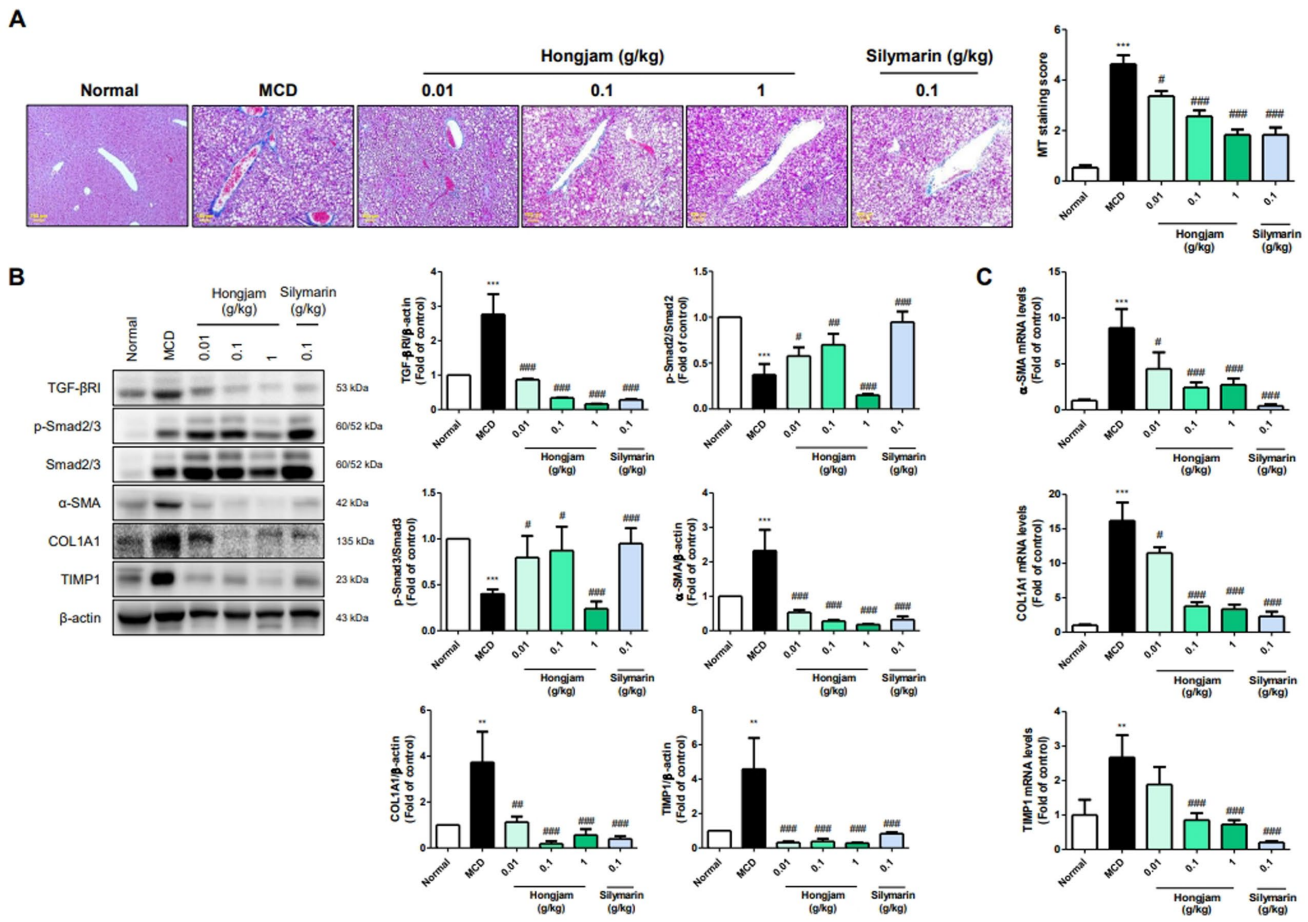
Hongjam suppresses hepatic fibrosis by targeting the TGF-β/Smad signaling pathway. **(A)** Masson’s trichrome–stained liver sections reveal collagen accumulation in MCD-fed mice, which is attenuated by Hongjam or silymarin. Fibrosis severity is quantified by histological scoring (scale bar = 100 μm; original magnification ×100). **(B)** Immunoblotting and densitometric quantification of fibrotic markers (TGF-βRI, p-Smad2, p-Smad3, α-SMA, COL1A1, TIMP1), normalized to total SMAD2/3 or β-actin. **(C)** qRT–PCR analysis of α-SMA, COL1A1, and TIMP1 transcripts, normalized to 18S rRNA. Data are presented as mean ± s.d. (*n* = 8 mice per group; Western blot and qRT–PCR quantification were performed using liver samples from *n* = 3 mice per group). Statistical significance was determined by one-way ANOVA followed by Tukey’s multiple comparisons test. **p < 0.01, ***p < 0.001 vs. normal group; #*p* < 0.05, ##*p* < 0.01, ###*p* < 0.001 vs. MCD group.

The anti-fibrotic effects of Hongjam were evaluated using MT staining, mRNA analysis of fibrosis-related markers, and Western blotting. The MCD diet-fed group exhibited extensive collagen deposition and marked fibrotic changes compared with the normal group. Hongjam treatment attenuated these changes in a dose-dependent manner, with the 1 g/kg group showing the greatest reduction (Figure 2A). TGF-β is a key mediator of hepatic fibrosis, primarily acting through activation of the TGF-β/Smad signaling pathway (1). To elucidate the molecular basis of Hongjam’s anti-fibrotic effects, we examined the activation state of this pathway (Figure 2B). The MCD diet significantly activated TGF-β signaling, as evidenced by increased TGF-β receptor I (TGF-βRI) protein levels. Hongjam treatment reduced TGF-βRI expression in a dose-dependent manner, indicating attenuation of pathway activation. Consistently, phosphorylated Smad2 (p-Smad2) and Smad3 (p-Smad3) levels were significantly decreased in the high-dose group (1 g/kg). The MCD diet also upregulated extracellular matrix (ECM)-related proteins, including α-smooth muscle actin (α-SMA), collagen type I alpha 1 (COL1A1), and tissue inhibitor of metalloproteinases 1 (TIMP1). Hongjam treatment markedly reduced the expression of these ECM components, supporting the suppression of fibrotic progression via inhibition of the TGF-β/Smad pathway. Furthermore, Hongjam downregulated fibrosis-related gene expression at the mRNA level. The MCD diet significantly increased mRNA expression of α-SMA, COL1A1, and TIMP1, while Hongjam suppressed these transcripts in a dose-dependent manner, with the most pronounced effects observed at 1 g/kg (Figure 2C). Collectively, these results demonstrate that Hongjam attenuates hepatic fibrosis in MCD diet-fed mice by inhibiting TGF-β/Smad signaling and downregulating fibrosis-related gene and protein expression, consistent with an anti-fibrotic effect in this model.

### 3.3 Hongjam attenuates dysregulated fatty acid oxidation and lipid metabolism in MCD diet-induced MASH

**Figure 3:**
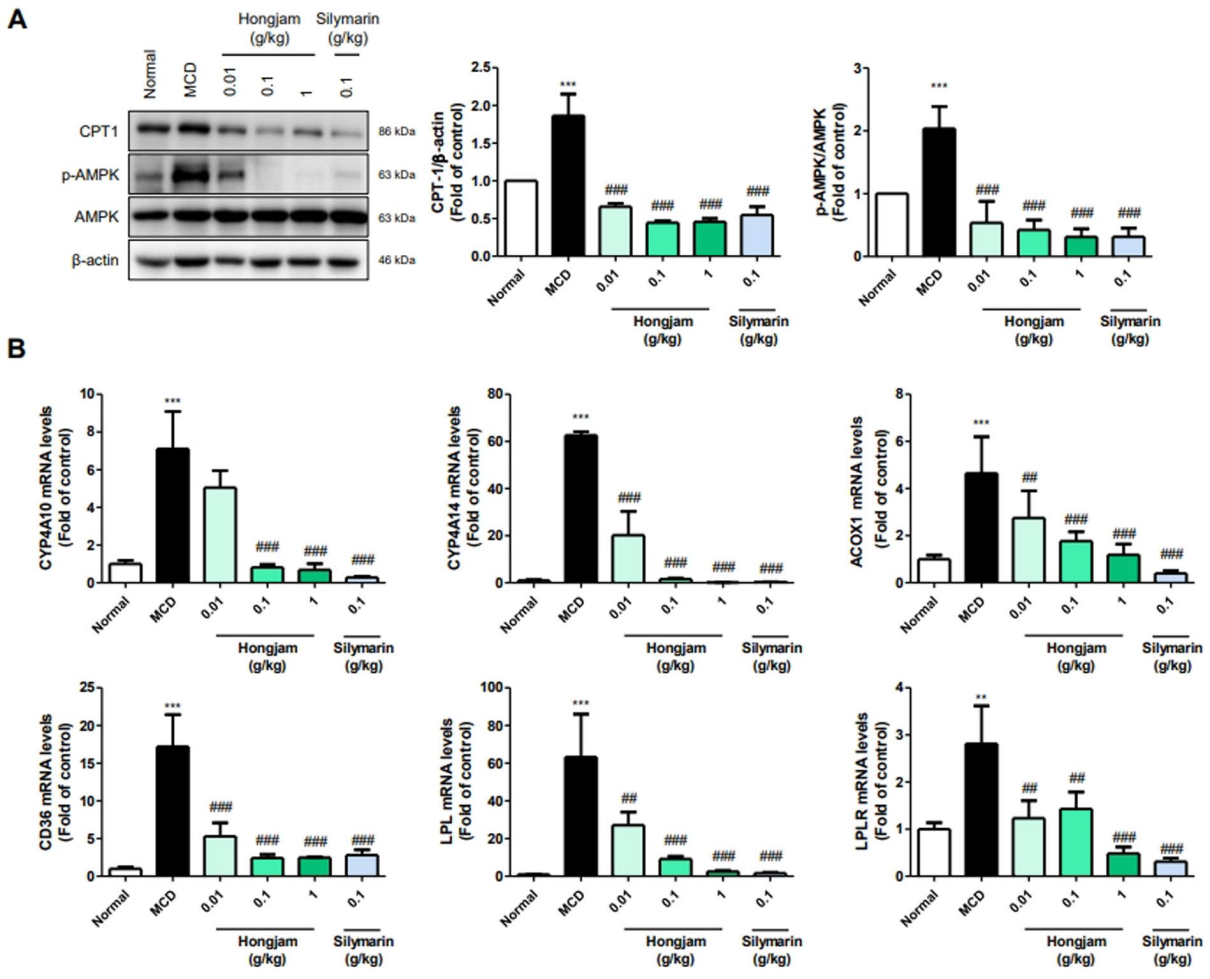
Hongjam rebalances dysregulated fatty acid oxidation and lipid metabolism in MCD diet-induced MASH. **(A)** Western blot analysis and quantification of CPT1 and phosphorylated AMPK in liver tissue, normalized to total AMPK or β-actin. **(B)** mRNA expression of lipid metabolism–related genes (*CYP4A10, CYP4A14, ACOX1, CD36, LPL, LDLR*) assessed by qRT–PCR and normalized to 18S rRNA. Data are presented as mean ± s.d. from three independent mouse liver samples per group (*n* = 3). Statistical significance was determined by one-way ANOVA followed by Tukey’s multiple comparisons test. *p* < 0.01, ***p < 0.001 vs. normal group; ##*p* < 0.01, ###*p* < 0.001 vs. MCD group.

AMPK is a key regulator of intracellular energy homeostasis (24, 25). The MCD diet induces hepatic lipid accumulation, inflammation, and fibrosis, thereby creating energetic stress. Consistent with this metabolic stress, an increase in phosphorylated AMPK (p-AMPK) was observed in the MCD group (Figure 3A). Carnitine palmitoyltransferase 1 (CPT1) expression was also elevated in the MCD group, indicating altered fatty acid metabolic signaling. Hongjam treatment attenuated these changes, suggesting partial restoration of fatty acid metabolic regulation. The MCD diet also significantly upregulated mRNA levels of cytochrome P450 family 4 subfamily A members 10 and 14 (CYP4A10 and CYP4A14), and acyl-CoA oxidase 1 (ACOX1) (Figure 3B), suggesting activation of microsomal and peroxisomal fatty acid oxidation pathways as compensatory responses. Additionally, genes involved in lipid uptake and transport, including cluster of differentiation 36 (CD36), lipoprotein lipase (LPL), and low-density lipoprotein receptor (LDLR), were markedly increased in the MCD group, reflecting disrupted lipid homeostasis. Hongjam suppressed these genes, with the strongest effect in the high-dose group, thereby promoting partial restoration of lipid metabolic balance. Collectively, these findings indicate that Hongjam alleviates MCD diet-induced metabolic dysregulation, accompanied by changes in key regulators of fatty acid oxidation and lipid metabolism. Notably, Hongjam partially normalized the MCD-associated increase in AMPK phosphorylation. Given that previous reports have suggested that increased AMPK phosphorylation in the MCD diet model may reflect hepatic energy stress associated with impaired fatty acid oxidation, mitochondrial injury, and ATP depletion, this partial normalization is consistent with an overall alleviation of metabolic stress in the MCD model (14).

### 3.4 Hongjam inhibits hepatic inflammation by downregulating NF-κB signaling and cytokine production

**Figure 4:**
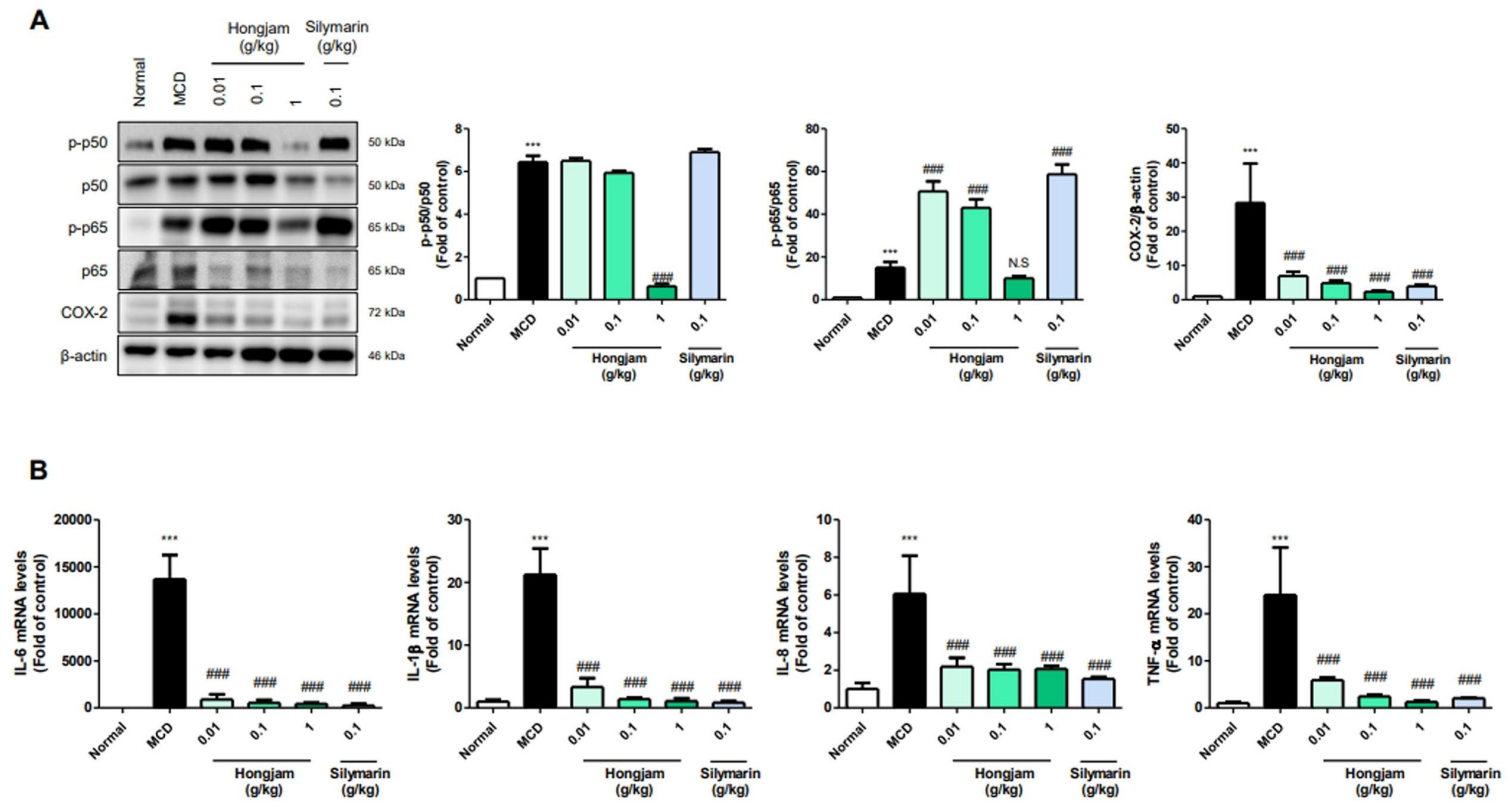
Hongjam inhibits hepatic inflammation by downregulating NF-κB signaling and cytokine production. **(A)** Western blots of phosphorylated and total p50, p65, and COX-2 in liver tissue, normalized to corresponding total forms or β-actin. **(B)** Relative mRNA levels of pro-inflammatory cytokines IL-6, IL-1β, IL-8, and TNF-α. Data are presented as mean ± s.d. from three independent mouse liver samples per group (*n* = 3). Statistical significance was determined by one-way ANOVA followed by Tukey’s multiple comparisons test. ***p < 0.001 vs. normal group; ###*p* < 0.001 vs. MCD group; n.s., not significant.

Hongjam treatment significantly reduced hepatic inflammation induced by the MCD diet by suppressing both protein and mRNA expression of key NF-κB–driven inflammatory mediators. In the MCD-fed group, phosphorylation of NF-κB subunits p50 and p65 was elevated, whereas Hongjam treatment led to a dose-dependent decrease in their phosphorylation (Figure 4A). Similarly, cyclooxygenase-2 (COX-2), an inflammatory enzyme strongly upregulated in the MCD group, was significantly downregulated in a dose-dependent manner following Hongjam administration. Notably, the high-dose Hongjam group (1 g/kg) showed reductions comparable to those observed in the silymarin-treated group, consistent with a strong anti-inflammatory effect (Figure 4A). Consistent with these protein-level changes, qRT–PCR analysis revealed robust, dose-dependent downregulation of key pro-inflammatory cytokines—including IL-6, IL-1β, interleukin-8 (IL-8), and TNF-α—in the livers of Hongjam-treated mice (Figure 4B). This coordinated suppression of NF-κB activation and its downstream effectors, including COX-2 and inflammatory cytokines, indicates that Hongjam effectively dampens the inflammatory cascade triggered by MCD feeding. Collectively, these findings demonstrate that Hongjam protects against MCD diet–induced hepatic inflammation by targeting NF-κB signaling and repressing multiple pro-inflammatory mediators.

### 3.5 Silk fibroin peptides suppress inflammatory signaling and modulate TGF-β/Smad-associated responses *in vitro*

**Figure 5:**
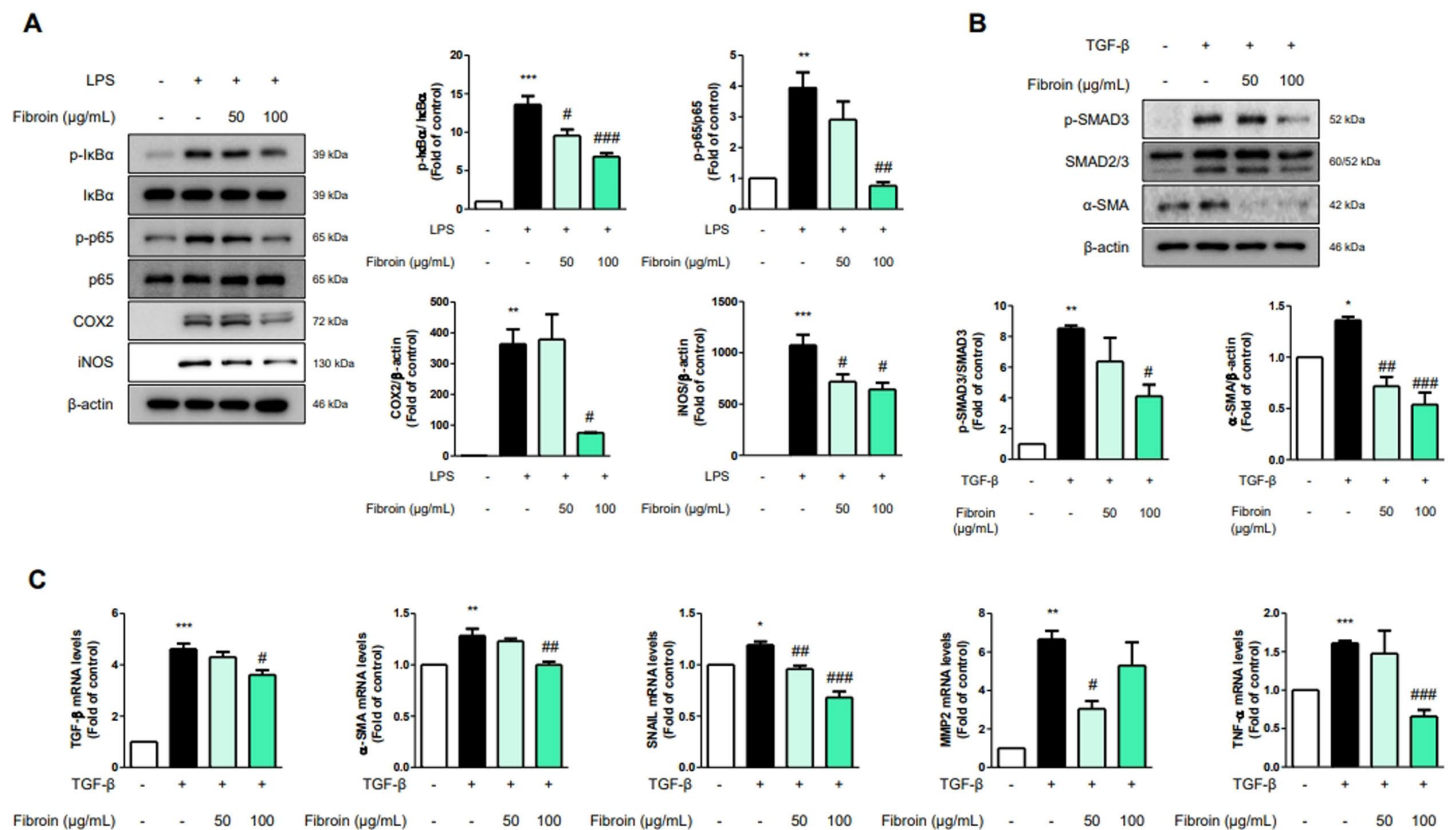
Silk fibroin peptides suppress inflammatory signaling and modulate TGF-β/Smad-associated responses *in vitro*. **(A)** In LPS-stimulated RAW264.7 cells, fibroin peptides reduce phosphorylation of IκBα and p65, and suppress COX-2 and iNOS expression. **(B)** In TGF-β–stimulated HepG2 cells, fibroin peptides attenuate p-SMAD3 and α-SMA protein expression. **(C)** qRT–PCR analysis shows reduced expression of TGF-β1, α-SMA, SNAIL, MMP2, and TNF-α transcripts following fibroin peptide treatment. Data are presented as mean ± s.d. from three independent experiments (each in triplicate). Statistical significance was determined by one-way ANOVA followed by Tukey’s multiple comparisons test. *p < 0.05, **p < 0.01, ***p < 0.001 vs. control (untreated); #*p* < 0.05, ##*p* < 0.01, ###*p* < 0.001 vs. LPS- or TGF-β–treated group.

To investigate the functional role of silk fibroin peptides, the putative active component of Hongjam, we conducted *in vitro* experiments using RAW 264.7 macrophages and HepG2 hepatocyte-derived cells. Silk fibroin treatment exhibited anti-inflammatory activity and modulated fibrosis-associated signaling by regulating key intracellular signaling pathways. In LPS-stimulated RAW 264.7 macrophages, silk fibroin significantly attenuated NF-κB–mediated inflammatory signaling. Western blot analysis revealed that LPS-induced phosphorylation of NF-κB subunits p65 and IκBα was dose-dependently suppressed following silk fibroin treatment (Figure 5A). In addition, expression levels of the inflammatory enzymes COX-2 and inducible nitric oxide synthase (iNOS) were markedly reduced, particularly at 100 μg/mL, consistent with suppression of LPS-induced inflammatory responses (Figure 5A). To assess whether silk fibroin peptides could modulate TGF-β/Smad-associated signaling, HepG2 cells were stimulated with TGF-β. TGF-β exposure led to robust activation of p-Smad3, a major downstream effector of the TGF-β/Smad pathway. Notably, silk fibroin treatment significantly decreased p-Smad3 levels, indicating attenuation of TGF-β/Smad signaling. Although HepG2 cells do not fully model hepatic stellate cell biology, TGF-β–induced Smad activation in hepatocyte-derived cells provides pathway-level insight into parenchymal stress responses associated with fibrogenic signaling. Consistently, α-SMA expression, a marker of fibrotic activation, was also downregulated (Figure 5B). Moreover, qRT–PCR analysis demonstrated that silk fibroin significantly suppressed the mRNA expression of fibrosis-associated genes upregulated by TGF-β in HepG2 cells, including TGF-β1, α-SMA, SNAIL, MMP2, and TNF-α. Among these, TGF-β1, α-SMA, and SNAIL were reduced in a dose-dependent manner, with significant suppression observed at 100 μg/mL (Figure 5C). Collectively, these findings indicate that silk fibroin peptides associated with Hongjam exert anti-inflammatory effects and modulate TGF-β/Smad-associated signaling responses *in vitro*.

## 4 Discussion

These findings support Hongjam, an edible insect-derived preparation, as a food-based candidate for liver health that may modulate multiple pathological processes relevant to MASH. We demonstrated that Hongjam, administered orally, alleviated hepatic injury and fibrosis in an MCD diet-induced mouse model of MASH, as evidenced by improvements in histology, plasma biomarkers, and pathway-level molecular alterations. The MCD diet model induces fibrosing steatohepatitis with histological features similar to human steatohepatitis and is widely used to investigate mechanisms of hepatic injury and fibrogenesis beyond simple steatosis (14, 26). Within this experimental framework, Hongjam consistently attenuated fibrosis, inflammation, and metabolic stress-associated signaling, suggesting an effect on multiple interrelated pathogenic processes that underlie MASH progression.

Fibrosis represents the strongest histological predictor of liver-related morbidity and mortality in patients with MASLD/MASH, surpassing steatosis and inflammatory activity as a determinant of long-term clinical outcomes (27). Despite substantial advances in understanding fibrogenic mechanisms, effective treatment options capable of reliably halting or reversing hepatic fibrosis remain limited, underscoring a major unmet clinical need (28). In this context, the observed anti-fibrotic effects of Hongjam are noteworthy. The MCD diet activated the TGF-ꞵ/Smad signaling axis, a central driver of hepatic stellate cell activation and extracellular matrix deposition during liver fibrogenesis (28). Hongjam treatment reduced collagen deposition and suppressed TGF-ꞵ receptor expression, Smad2/3 phosphorylation, and downstream fibrogenic gene and protein expression, supporting pathway-level inhibition of fibrotic remodeling rather than nonspecific histological improvement.

Chronic inflammation is another core component of MASH pathogenesis and contributes directly to both hepatocellular injury and fibrosis progression (29). NF-κB functions as a central transcriptional regulator linking cellular stress to inflammatory cytokine production in steatohepatitis (29). Consistent with this paradigm, MCD feeding increased NF-κB activation and upregulated pro-inflammatory mediators, including COX-2, iNOS, IL-6, IL-1ꞵ, and TNF-α, whereas Hongjam treatment dose-dependently suppressed NF-κB phosphorylation and downstream inflammatory gene expression. Although this study focuses on MASH, NF-κB-driven inflammation is a conserved feature across chronic liver diseases, including alcoholic liver disease, in which gut-derived inflammatory stimuli can promote hepatic inflammation through the TLR4/NF-κB axis (30). Given the established role of persistent NF-κB-driven inflammation in amplifying profibrotic signaling, inhibition of this inflammatory axis likely contributes upstream to the attenuation of fibrotic remodeling observed in Hongjam-treated mice.

Metabolic stress represents a third, tightly interconnected axis of MASH pathophysiology (31). The MCD diet disrupts hepatic lipid handling and induces mitochondrial dysfunction, leading to ATP depletion and activation of energy stress responses in hepatocytes (14). In line with this, MCD feeding increased AMPK phosphorylation and altered the expression of genes involved in fatty acid oxidation and lipid uptake, suggesting a marked metabolic stress response (32). Hongjam normalized the MCD-associated increase in AMPK phosphorylation and lipid metabolism-related gene expression, consistent with an overall alleviation of metabolic stress. Consistent with our findings, some MCD diet-induced MASH/NASH studies using naturally derived interventions have reported increased hepatic p-AMPKα following MCD feeding, with subsequent attenuation after treatment (33, 34). Together with these reports, our findings support the interpretation that reduced AMPK phosphorylation may accompany improvement of MCD-associated metabolic stress. Nevertheless, a clear limitation of the present study is that AMPK activity was not directly manipulated. Therefore, further functional assays, together with direct lipid quantification and mitochondrial functional analyses, will be required to clarify whether changes in AMPK phosphorylation play a causal role in Hongjam-mediated hepatoprotection and whether these molecular changes translate into improved hepatic energy homeostasis. It should be noted that the MCD model does not fully recapitulate the metabolic features of human MASLD, including obesity and insulin resistance, and therefore primarily reflects inflammatory and fibrotic aspects of disease progression.

Notably, the concurrent modulation of metabolic stress (AMPK-associated signaling), inflammatory activation (NF-κB), and fibrotic remodeling (TGF-ꞵ/Smad) positions Hongjam as a multi-axis modulator rather than a pathway-selective intervention (28). This distinction is mechanistically relevant because these processes form self-reinforcing feed-forward loops, wherein metabolic stress promotes inflammation, inflammation accelerates fibrosis, and fibrosis further aggravates hepatic dysfunction (35). Accordingly, therapeutic strategies that target a single signaling axis may be insufficient to interrupt disease progression due to compensatory network responses (31). Recent pharmacologic advances in MASH have focused on discrete pathogenic axes (36). Semaglutide (Wegovy), a GLP-1 receptor agonist, improves steatohepatitis primarily through systemic metabolic effects such as weight loss and improved insulin sensitivity, but has shown inconsistent or limited efficacy in inducing fibrosis regression in clinical trials (36). Resmetirom (Rezdiffra), a selective thyroid hormone receptor-ꞵ agonist, directly targets hepatic lipid metabolism and reduces liver fat content, but its therapeutic action is largely centered on metabolic correction, with relatively modest effects on inflammatory and fibrotic signaling (37). In contrast, Hongjam directly suppressed fibrogenic and inflammatory pathways most closely linked to disease progression while simultaneously modulating metabolic stress-associated signaling, a mechanistic profile that may be relevant in a multifactorial disease such as MASH.

From a translational perspective, the administration of Hongjam warrants consideration of dose feasibility (38). Based on body surface area conversion, the highest dose used in this study (1 g/kg in mice) corresponds to an estimated human equivalent dose of approximately 4.9 g/day for a 60-kg adult, a range potentially feasible for oral administration in humans (38). However, translation to human application will require standardization of active components, assessment of oral bioavailability, and pharmacokinetic-pharmacodynamic linkage to define effective and scalable dosing.

In interpreting these findings, the compositional nature of Hongjam merits careful consideration. Hongjam is a complex natural preparation containing multiple bioactive constituents, including silk fibroin- and sericin-derived peptides, unsaturated fatty acids, and micronutrients, each of which has been reported to exert antioxidant, anti-inflammatory, or metabolic regulatory activities in prior studies (17). Rather than acting through a single dominant compound, Hongjam is therefore likely to exert its biological effects through composite or synergistic actions across multiple components (39). Among these constituents, fibroin-associated peptides have attracted attention due to their repetitive Gly-Ala-Ser-rich motifs and reported capacity to modulate stress-responsive and inflammatory signaling pathways (17, 39). In line with this, our *in vitro* data demonstrate that fibroin peptides suppress NF-κB-mediated inflammatory signaling in macrophages and modulate TGF-β/Smad-associated signaling responses in HepG2 hepatocyte-derived cells, supporting their functional relevance within the broader activity profile of Hongjam. However, because HepG2 cells do not represent the primary effector cells of liver fibrosis, these in vitro data should be interpreted as pathway-level evidence of TGF-β/Smad signaling modulation rather than as direct evidence of fibrosis inhibition in hepatic stellate cells. Future studies using hepatic stellate cell-based models, including primary HSCs or established HSC cell lines, will be required to directly validate the anti-fibrotic relevance of Hongjam-derived silk fibroin peptides. In addition, because Hongjam was administered as a whole preparation *in vivo*, the relative contribution of individual components cannot be definitively resolved in the present study and will require future investigation using component-resolved or fraction-based approaches. Moreover, the present study did not determine whether fibroin-derived peptides are orally absorbed, reach the liver, or achieve effective concentrations in vivo, and pharmacokinetic and biodistribution analyses will be required to address this issue. Although causality cannot be definitively established in vivo in the present study, the concordant suppression of NF-κB and TGF-β/Smad signaling by fibroin peptides supports their potential functional relevance within the broader activity profile of Hongjam.

Notably, whereas our previous work in high-fat diet models characterized Hongjam as a metabolic regulator that improves steatosis and insulin resistance, the present study demonstrates that Hongjam also directly targets inflammatory and fibrotic signaling cascades in a stringent MCD model of advanced MASH. This broadens the functional profile of Hongjam as a food-derived preparation, suggesting its potential utility as a dietary adjunct that extends from preventive metabolic modulation to anti-inflammatory and anti-fibrotic effects relevant to disease modification. Given that Hongjam is an edible food-derived preparation traditionally consumed in East Asia, its demonstrated multi-axis modulation of metabolic stress, inflammation, and fibrogenic signaling suggests potential relevance not only as a pharmacological candidate but also as a functional dietary component for liver health. These findings support further investigation into the role of insect-derived functional foods as nutritional strategies targeting metabolic liver diseases.

In this research, Hongjam attenuated MCD diet-induced MASH by concurrently suppressing TGF-ꞵ/Smad-driven fibrogenesis, inhibiting NF-κB-dependent inflammatory programs, and normalizing metabolic stress-associated signaling. Given that fibrosis remains the principal determinant of clinical outcomes and effective anti-fibrotic therapies are limited, Hongjam represents a potential natural product-derived candidate with disease-modifying activity. Moreover, its multi-target mechanism suggests that Hongjam could complement metabolism-focused agents such as GLP-1 receptor agonists or thyroid hormone receptor-ꞵ agonists, supporting its potential utility as an adjunctive component of combination therapeutic strategies for MASH.

## Data Availability

The original contributions presented in this study are included in the article and Supplementary material. Further inquiries can be directed to the corresponding author.
