# Supplementary material for: Hongjam, an edible silkworm-derived food, attenuates steatohepatitis and fibrosis via multi-axis modulation of metabolic stress, inflammation, and fibrogenic signaling
Source: Front Nutr. 2026 May 26;13:1839551. doi: 10.3389/fnut.2026.1839551 (PMC13246375; doi:10.3389/fnut.2026.1839551)

Supplementary Fig. S1. Raw uncropped western blot images corresponding to Fig. 2

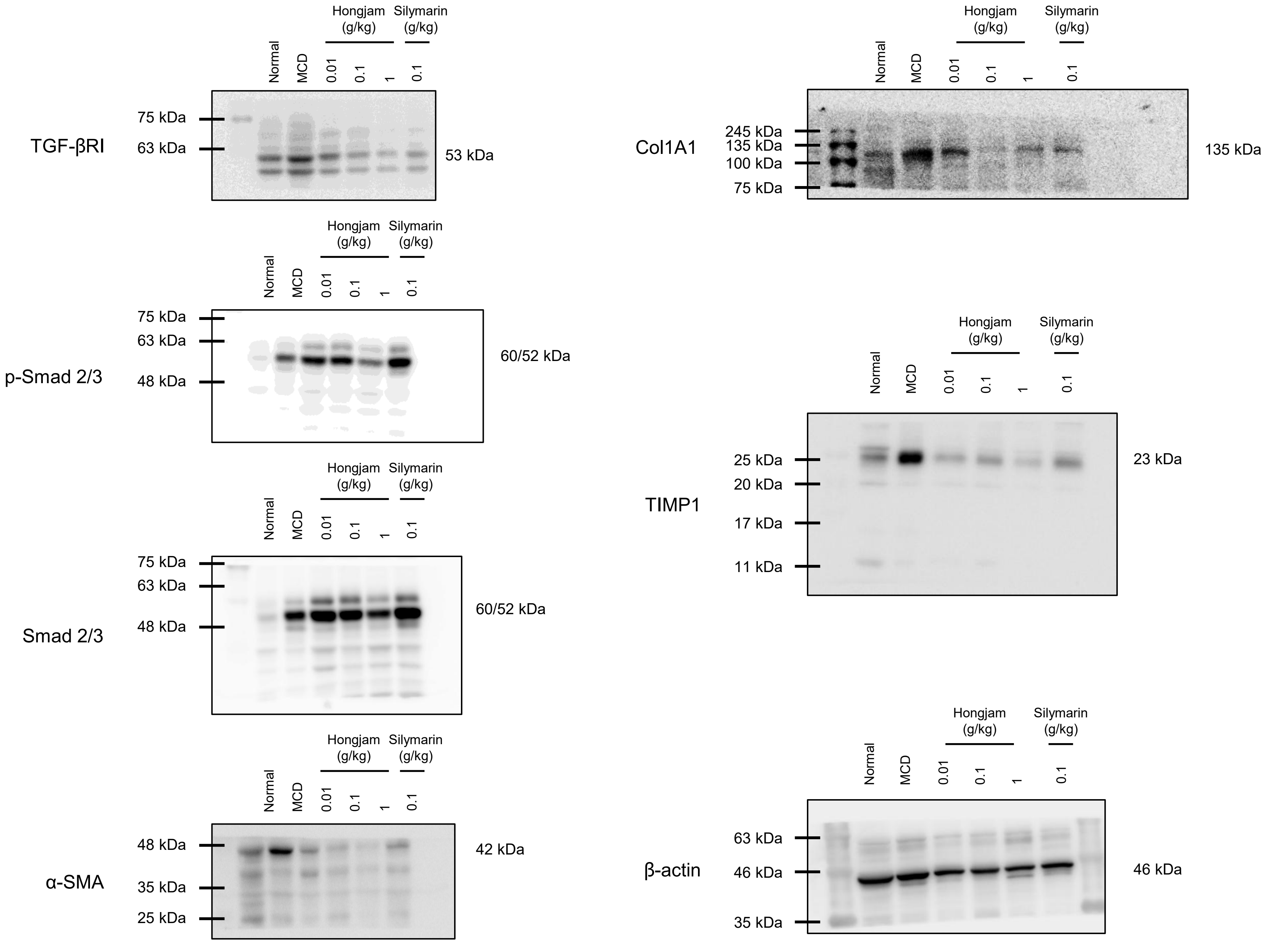

Supplementary Fig. S2. Raw uncropped western blot images corresponding to Fig. 3

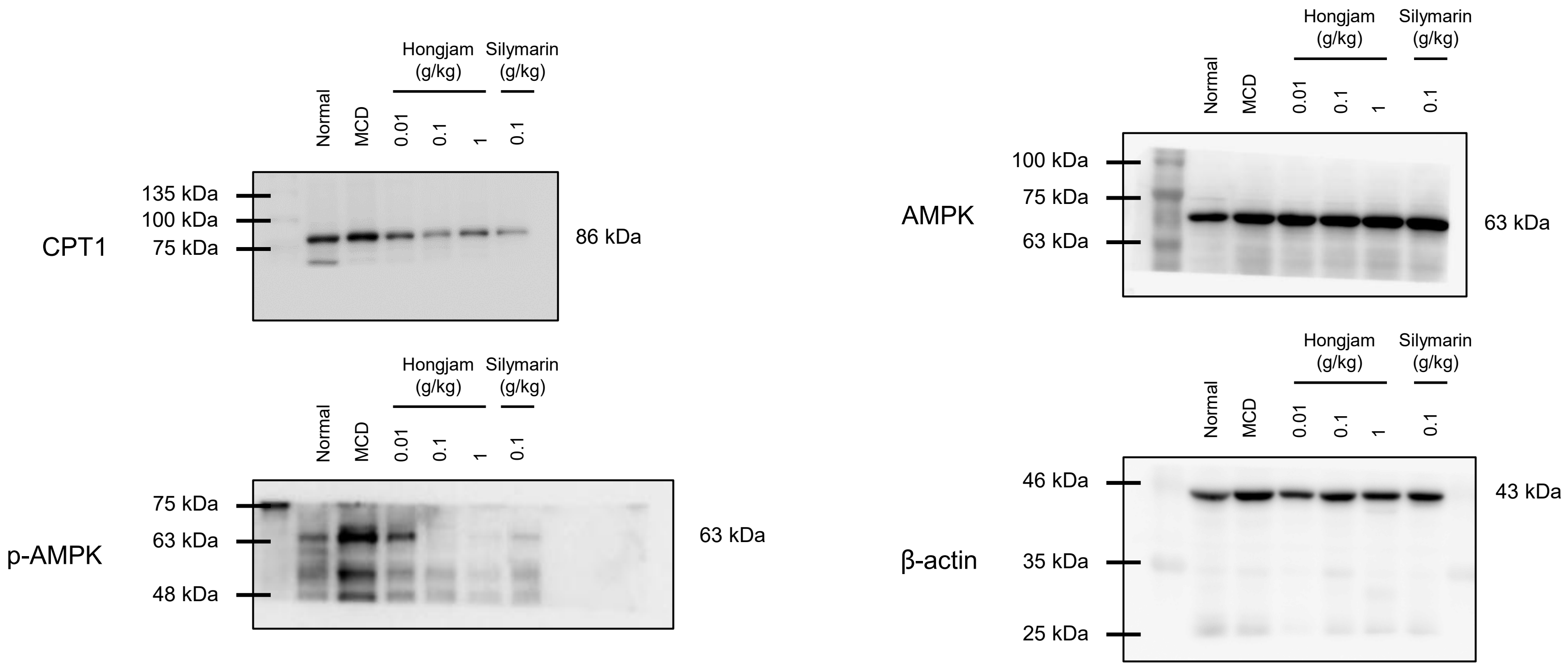

Supplementary Fig. S3. Raw uncropped western blot images corresponding to Fig. 4

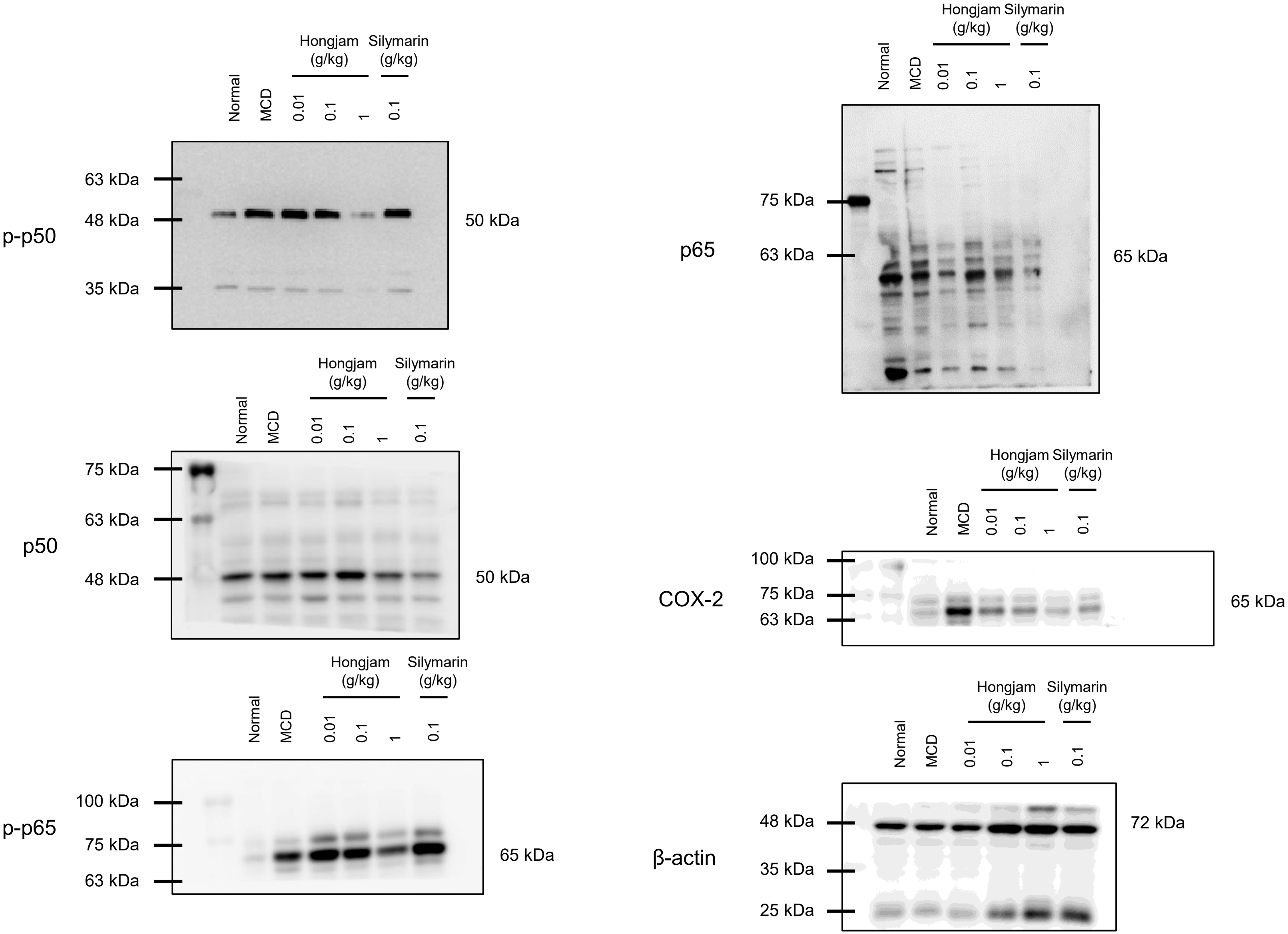

Supplementary Fig. S4. Raw uncropped western blot images corresponding to Fig. 5A

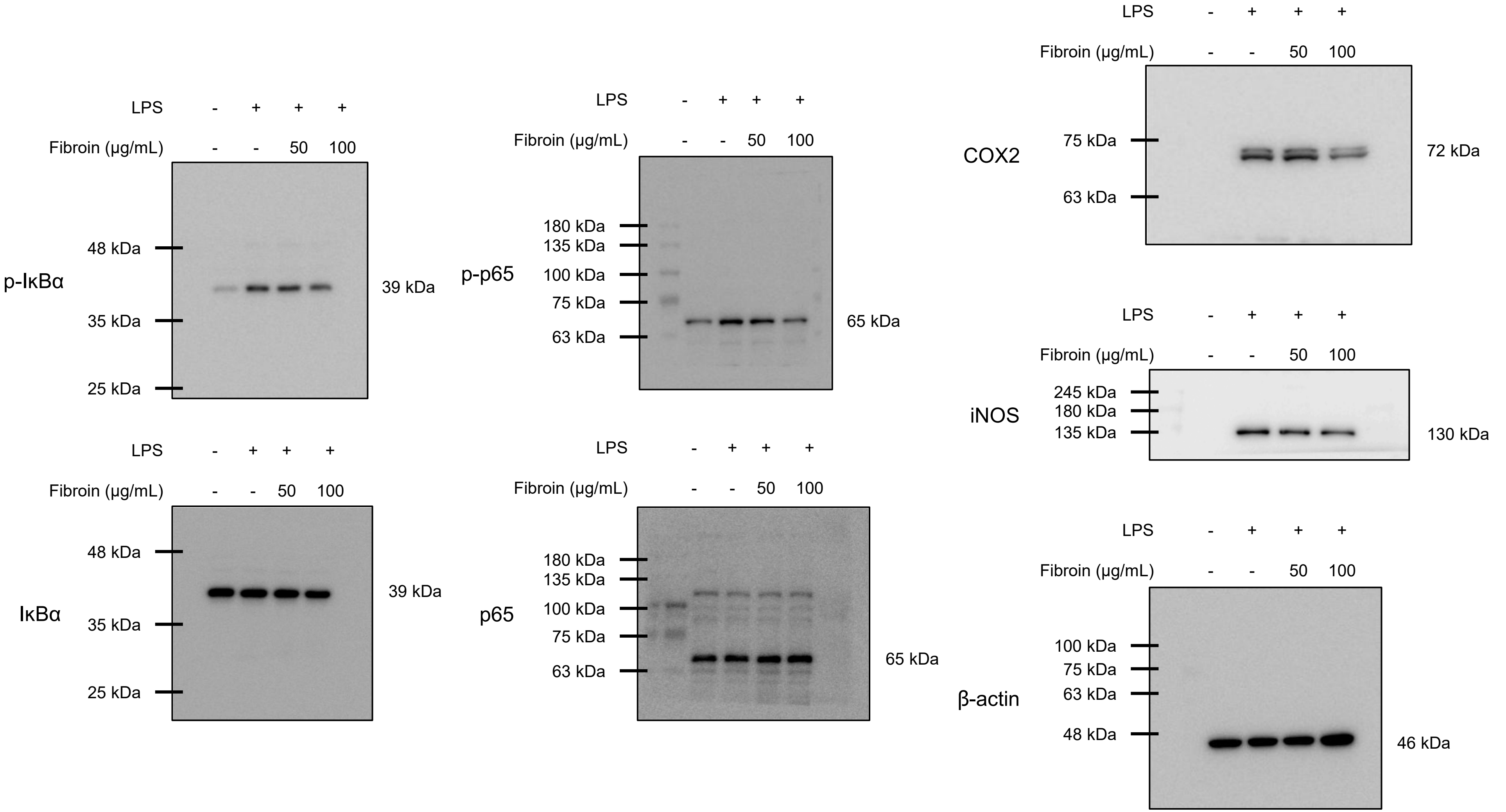

Supplementary Fig. S5. Raw uncropped western blot images corresponding to Fig. 5B

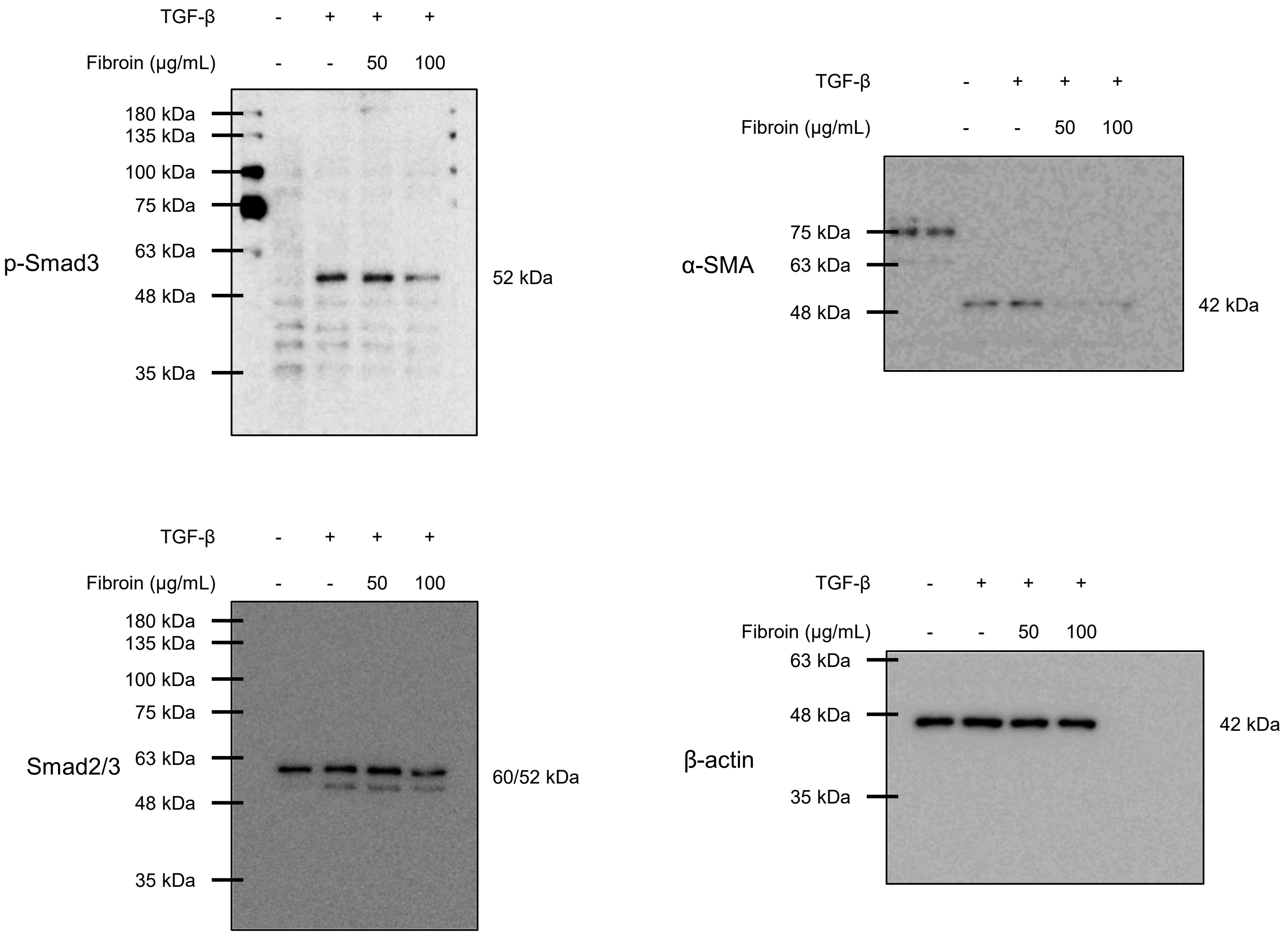

Supplement: Supplementary file 1 [file Data_Sheet_1.PDF]
