# Supplementary material for: Hongjam, an edible silkworm-derived food, attenuates steatohepatitis and fibrosis via multi-axis modulation of metabolic stress, inflammation, and fibrogenic signaling
Source: Front Nutr. 2026 May 26;13:1839551. doi: 10.3389/fnut.2026.1839551 (PMC13246375; doi:10.3389/fnut.2026.1839551)
Supplement: Supplementary file 2 [file Table_1.DOCX]

**Table S1. The nutrient composition of Hongjam**

| **Nutrient (unit)** |  | |
| --- | --- | --- |
| Amino acids (%) | Glycine | 12.335 |
|  | Alanine | 9.778 |
|  | Serine | 6.757 |
|  | Aspartic acid | 4.679 |
|  | Tyrosine | 4.560 |
|  | Glutamic acid | 3.824 |
|  | Valine | 2.399 |
|  | Lysine | 2.193 |
|  | Threonine | 2.185 |
|  | Leucine | 1.983 |
|  | Arginine | 1.958 |
|  | Phenylalanine | 1.746 |
|  | Isoleucine | 1.214 |
|  | Proline | 1.148 |
|  | Histidine | 1.083 |
|  | Methionine | 0.644 |
|  | Cysteine | 0.427 |
|  | Tryptophane | 0.425 |
| Fatty acids (%) | Myristic acid (C14:0) | 0.17 |
|  | Palmitic acid (C16:0) | 24.49 |
|  | Stearic acid (C18:0) | 8.55 |
|  | Oleic acid (C18:1n-9) | 31.22 |
|  | Palmitoleic acid (C16:1n-7) | 0.86 |
|  | Linolenic acid (C18:3n-3) | 27.86 |
|  | Linoleic acid (C18:2n-6) | 6.50 |
|  | Eicosenoic acid (C20:1n-9) | 0.23 |
|  | γ-Linoleic acid (C18:3n-6) | 0.13 |
|  | Vaccenic acid (C18:1n-7) | 0.00 |
|  | Eicosatrienoic acid (C20:3n-3) | 0.00 |
|  | Eicosapentaenoic acid (C20:5n-3) | 0.00 |
|  | Docosatetraenoic acid (C22:4n-6) | 0.00 |
|  | Docosahexaenoic acid (C22:6n-3) | 0.00 |
|  | Total | 100.00 |
|  | ▪ Saturated fatty acids | 33.21 |
|  | ▪ Unsaturated fatty acids | 66.79 |
|  | ▪ Mono-unsaturated | 32.31 |
|  | ▪ Poly-unsaturated | 34.49 |
| Proximal analysis  (g/100 g) | Water | 2.85 |
|  | Crude protein | 68.66 |
|  | Crude fat | 10.99 |
|  | Crude ash | 3.26 |
|  | Crude fiber | 1.77 |
| Vitamins | Vitamin A (μg/100 g) | 576.80 |
|  | Vitamin B1 (mg/100 g) | 0.26 |
|  | Vitamin B2 (mg/100 g) | 3.67 |
|  | Vitamin B6 (mg/100 g) | 0.35 |
|  | Vitamin B12 (μg/100 g) | 0.00 |
|  | Niacin (mg/100 g) | 1.28 |
|  | Folic acid (mg/100 g) | 0.40 |
|  | Biotin (μg/100 g) | 0.00 |
|  | Vitamin C (mg/100 g) | 12.62 |
| Polyphenols (mg/100 g) | Flavonoids | 380.16 |
| Minerals (mg/100 g) | Potassium (K) | 1045.4 |
|  | Phosphorus (P) | 688.27 |
|  | Sulfur (S) | 326.45 |
|  | Magnesium (Mg) | 199.25 |
|  | Calcium (Ca) | 198.90 |
|  | Sodium (Na) | 14.93 |
|  | Zinc (Zn) | 5.76 |
|  | Iron (Fe) | 3.17 |
|  | Manganese (Mn) | 1.60 |
|  | Copper (Cu) | 1.20 |
|  | Chromium (Cr) | 0.00 |
|  | Lead (Pb) | 0.00 |
|  | Cadmium (Cd) | 0.00 |
|  | Mercury (Hg) | 0.00 |
|  | Arsenic (As) | 0.00 |
